# Influence of Increasing Task Complexity and Use of Informational Assistance Systems on Mental Workload

**DOI:** 10.3390/brainsci11010102

**Published:** 2021-01-14

**Authors:** Dominic Bläsing, Manfred Bornewasser

**Affiliations:** 1 Institute of Psychology, University Greifswald, Franz-Mehring-Str. 47, 17489 Greifswald, Germany; bornewas@uni-greifswald.de; 2 Institute for Community Medicine, Prevention Research and Social Medicine, University Medicine Greifswald, Walther-Rathenau-Str. 48, 17489 Greifswald, Germany

**Keywords:** mental workload, human–machine interaction, manual assembly, task complexity, ECG, eye-tracking

## Abstract

(1) Background: Cognitive aspects and complexity in modern manual mixed model assembly are increasing. To reduce mental workload (MWL), informational assistance systems are introduced. The influence of complexity and used assistance system on MWL should be investigated to further improve the implementation of such assistance systems. (2) Methods: Using a simulated close to real-life assembly task a 2 × 3 design was chosen, with two levels of assembly complexity (within subjects) and three different assistance systems (paper, Augmented Reality (AR)-glasses, tablet–between subjects). MWL was measured using either physiological response (electrocardiogram (ECG) and eye-tracking) or performance indicators. (3) Results: An influence of task complexity on MWL can be shown. Additionally, usability based differences between the used assistance systems become more evident with reference to the results of area of interest analysis. (4) Conclusions: Using a multi-modal measurement approach, it is possible to detect complexity-based differences in MWL. Additional research on validity and alignment is needed to further use these for (neuro-) ergonomic considerations and recommendations.

## 1. Introduction

### 1.1. Modern Assembly Implies Increase of Complexity

Increasing mass customization in assembly processes leads to a higher overall work complexity. Where assembly workers formerly had to repeatedly and routinely assemble homogeneous products, nowadays, so called mixed model assembly is taking place resulting in heterogeneous product variants. As a consequence, the mental aspects of assembly work have gained importance through higher entropy in the assembly line connected to higher choice density during the assembly process. The overall complexity of a specific assembly work process results from the sum of choices to be made over all steps across the variants in a limited time.

Every single choice is based on an episode of information processing including aspects of attention, interference control, inhibition, and behavioral execution. The more information is available in the assembly system, the more cognitive activity is needed in order to cope with the uncertainty of the worker associated with each choice and each behavioral execution. Operator choice complexity is defined as the mean uncertainty or randomness (of the product) in a series of different product variants that require changing choice processes and behavioral adaptations over a certain period of time, thus leading to higher mental workload [1]. It is the aim of the present study to show that higher operator choice complexity in concrete manual assembly tasks leads to increased mental workload. Additionally, the relationship of different informational assistance systems on mental workload will be shown.

### 1.2. Information Processing Is Associated with Mental Workload

Research on choice behavior has a long tradition in cognitive psychology. There are some parallels to reaction time studies [2]. As empirically shown, the response times of correct responses to a stimulus increase either with an increasing number of stimuli or reaction alternatives to be considered. The more choices are required and the more alternatives have to be taken into account, the greater the complexity becomes and more time is required for the correct completion of the task.

Further confirmation comes from perception research. Using eye tracking to gain insight into gaze behavior (fixations and saccades), it is possible to make assumptions over the perceptive and cognitive base of choice processes [3,4]. With higher task complexity and an increasing number of choices, there is a necessity for repeated and longer fixation of instructions. Through those longer and repeated fixation processes, informational noise is reduced, stress on the working memory is relieved, and new capacities are made available. Both lines of research show that the structure and number of cognitive processes are determined by the interplay of task features, operator resources, and work conditions.

### 1.3. Mental Workload as a Theoretical Construct

Mental workload (MWL) can be seen as the ratio between environmental and task relevant demands and the internal supply of mental resources. MWL is related to cognitive processes, starting from information intake and processing and ending with motor response initialization and motor pattern monitoring [5]. It is bottlenecked by limited capacities, especially in working memory [6,7]. Each choice process can be seen as a cognitive operation characterized by resource usage in specific, connected brain areas [8]. With increasing task demands, resource usage also increases and more available capacity is used. Thus, MWL describes “how hard the brain is working to meet task demands” [9]. This implies the question about individual, absolute, or relative capacity limits.

It is assumed that a task is mentally more demanding if there is no cognitive automatism available to master it. Thus, the demand–supply ratio will become unbalanced (more demand than supply) and a state of mental overload can occur. During work tasks, demand and supply are constantly changing through allostatic and adaption processes. If the point of mental overload is reached, an adaption is no longer possible, resulting in a stressful state and decreasing performance. Young et al. [10] discussed the dynamically changing relationship between demand, supply, and performance in their ergonomically characterized red-lines-model of mental workload, which has similarities to the maximal-adaptability-model presented by Hancock and Warm [11]. Modern medical-neurological approaches try to give detailed references about the cortical structures and neurophysiological processes underlying information processing [12,13].

From a more practical ergonomic point of view, the main task is to identify when mental workload becomes suboptimal and will lead to errors and incidents as well as to recommend appropriate relief measures. The central aim of these measures is a reduction of information access costs [14], for example, when information presentation is diversified by using different resource modalities [5]. Promising attempts have been made in the field of human–machine interaction to automatically detect mental overload using machine learning algorithms and to relieve strain by emitting specific tasks to the machine and release tied-up resources [15].

### 1.4. Informational Assistance Systems in Mixed Model Assembly

In mixed model assembly systems, there is a trend to integrate informational assistance systems to reduce mental load during processes with high choice density by either offering an informational guidance (e.g., pick-by-light or put-to-light systems for part picking [16]) or stepwise, and even individually oriented, assembly instructions. Thus, informational assistance systems can be seen as “cognitive amplifiers” [17]. In parallel to exoskeletons that strengthen physical action, they enhance cognitive abilities to process information by easing the information access and keep working memory capacity free to use. Matthews et al. [18] called this approach augmented cognition. A similar effect can be obtained if the structure of information presentation is compatible with the inherent mental model of the worker [19,20]. An abstract construction plan might fit the engineer’s mental model, but can put additional strain on the assembly worker who only needs a few details or some handling advices. Compatibility helps to avoid time consuming interpretation difficulties. Overall, the usage of informational assistance systems should not cause an increase in information processing connected to additional (mental) strain.

Modern informational assistance systems are usually digitally supported, often using picture-text combinations on tablets or AR-glasses [21]. However, traditional paper based approaches can lead to comparable reductions in workload. Content and structure of information are decisive, and the medium itself is less important. Both aspects should be separated. When content and structure of instructions are identical for different assistance systems like paper, tablets, or AR-glasses, no differences in mental workload should occur. However, possible noticeable differences should be caused through the assistance system’s individual usability (for a negative example using head-mounted displays, see [22]). Aside from the ease of use, the workers’ familiarity with the used device and the integration of the assistance system into the working place are important parts of usability [23,24].

### 1.5. Mental Workload and the Problem of Robust Quantification

For a better understanding of a theoretical concept like MWL, it is necessary to find ways to operationalize it. Using physiological and neurophysiological indicators like changes in brain activity, patterns of oxygenated and deoxygenated blood in different brain areas or pupillary response will make MWL a construct with a close real world connection ([12] for a deeper view). This operationalization will help to understand “how the brain carries out the complex tasks of everyday life” or “how the brain processes visual, auditory and tactile information” [25]. Understanding such processes is the necessary base to derive specific work place related recommendations to improve (neuro-) ergonomics. In addition to Parasuraman’s overall approach of cognitive neuroergonomics under the slogan “the brain in action and at work” [25], Benarroch [26] proposed a central autonomous network, which is seen as a component of an internal regulation system through which the brain controls visceromotor, neuroendocrine, and behavioral responses. This approach focuses on the close connection between the central nervous system (CNS) and autonomous nervous system (ANS) not only for controlling motoric responses, but also bio-physiological changes due to changing situations and the overall process of allostasis [27].

There are several possible methods and even more indicators to quantify MWL during work processes [18,28,29]. Indicators vary in their spatial and temporal resolution and are only able to show a specific part of mental workload, which lowers their overall validity. Measuring MWL in a multimodal manner seems to be an appropriate solution for this problem [18,30,31,32]. Methods for MWL assessment can be categorized in subjective, performance based, and physiological measurements with an increasing tendency for wearable, mobile, and non-invasive neurophysiological methods like eye-tracking, EEG, or fNIRS [33,34,35]. Compared to performance based data and subjective ratings, physiological methods offer a continuous data stream and thus offer chances for live analysis using machine learning algorithms and a direct approach to identify task related changes in MWL (for example, using heart rate (HR), see [36]).

### 1.6. Mental Workload Indicators Used in this Study

To quantify MWL two different physiological measurement methods were chosen: electrocardiography (ECG, heart rate (HR), and heart rate variability (HRV)) and eye movement assessment (pupillary response, fixation duration, saccadic peak velocity, and area of interest analysis). In addition, performance related indicators (time, failure) are used.

Cognitive functions like selective attention, response selection, inhibition of prepotent responses, and executive control are all thought to be dependent on working memory, the neurological basis of which are neural network connections between autonomic (ANS) and central nervous system (CNS), especially during the regulation of deliberate, purposeful behavior, mental load, and allostatic reactions [27,36,37]. The inhibitory characterized connection can also be called the central autonomic network [26]. It influences HR and HRV via various cortical and subcortical levels using the stellate ganglion and vagus nerve [38]. This network is associated with the processes of response organization and selection and serves to control psychophysiological resources in attention, working memory, and behavioral adaptation. It allows for maximal organism flexibility in rapid adaption to changing environmental demands. If this circuit is blocked, the ability to recruit and utilize neural support to meet a particular demand is hampered and the organism is thus less adaptive. Increased cognitive activity leads to changes in the homeostatic balance and therefore allostatic adjustments, which will change the distance between R-peaks and therefore the HR and HRV parameters. HRV is not only seen as an indicator for ANS balance, but also for central–peripheral neural feedback and CNS-ANS integration [39].

For this study, all relevant electrocardiographic measures are based on temporally distances between consecutive heartbeats. HR is chosen as an overall indicator for arousal [40], RMSSD (root mean sum of squared distance) as a time-based HRV parameter with focus on parasympathetic activity [39], and rrHRV (relative RR intervals) as an additional HRV parameter that is more robust against changes in overall HR and movement artifacts [41].

Eye tracking seems to have a closer connection to CNS activity, being cited as the window to the soul by Kahneman [42], or eye movements as a window to perception and cognition by McCarley and Kramer [43]. Using eye and gaze related parameters, it seems to be possible to gain insight into information intake and processing. Eye movement regulation, as a deliberate and not spontaneous act, is situated in the dorsolateral prefrontal cortex, which is closely connected to working memory, attention, and interference control [13,44,45]. Therefore, measurement of oculomotor data is a useful complement to ECG related parameters, which focus more on executive functions of response selection and the performance of complex tasks.

Various physiological indicators for eye and gaze movements are available with different findings regarding their relationship to MWL. This study will focus on the most important indicators: pupillary response (PR), fixation duration (FD), and saccadic peak velocity (SPV) (for an overview, see [43]). With increasing cognitive or emotional load, pupil size (measured as PR) will increase [46,47,48,49]. Normal eye movements are a consecutive sequence of fixations and short volatile saccades. Fixation duration can be seen as an indicator for information intake (longer fixations–more informational load) [50,51] while indicators based on saccades (like amplitude, direction, duration, and peak velocity [52]) have a closer connection to the overall mental load (for an overview, see [4]).

Particularly in stressful situations, fixation duration behavior for stress inducing objects can change toward shorter fixations [51]. This more avoidance orientated behavior can be analyzed using area of interest (AOI) analysis. AOI analysis offers insight into individual gaze behavior which has a close connection to the operator’s mental model of a given task [53]. Using the absolute or relative number of fixations for all given areas can show which ones are more or less used to perform a specific task [54]. Further used parameters are the gaze or dwell time (summed up time of all fixations in a specific area) [55], number of revisits (returns to the specific area), and the average fixation duration.

### 1.7. Principal Conclusions and Aim of the Study

The main aim of this study was to show the connection between the complexity of different manual assembly tasks and MWL under time pressure in a natural setting. Additionally, it was intended to demonstrate that the usage of the same instructional material on different informational assistance systems will create equal mental workload. This should demonstrate that the used instructions are more important than the used system. Potentially occurring differences in MWL will be a result of differences in system usability (like hands-free solutions or having information always available in the field of view).

To do so, it is necessary to use objective physiological measures of MWL to detect changes with a higher granularity. A neurological consideration of the “brain at work” as Parasuraman [25] proposed can change the way risk and workload assessments will be performed in the future. This is only possible as long as experimental studies can corroborate these assumptions. The following hypotheses will be tested:

**H1:**  *Complexity in mixed model assembly tasks comes along with increased information processing activities and various choice situations. An increase of complexity implies an increase of choices. The more choices there are, the more MWL will occur. Thus, between levels of complexity there are differences in MWL to be predicted (performance and physiologically based indicators).*

**H2:**  *Informational assistance systems can help to reduce MWL. To do so, it is important to show just the necessary information at the right space, at the right time [17,56]. Under the assumption of equal usability, the presentation of identical instructions for each tested informational assistance system will not result in significant differences in task performance and MWL.*

**H3:**  *Increasing assembly complexity stimulates a need for more information integration. This implies a changing gaze behavior. Object-related gaze behavior will change to more interactions with the assistance system and the assembly object, accompanying an increase in fixation durations. Simultaneously, learning processes and experience will lead to reduced dwell times on assembly parts and tools. Potential differences between the used information assistance systems will be usability based.*

## 2. Materials and Methods

### 2.1. Experimental Design

To gain insights into the relationship between complexity, used assistance systems, and mental workload, a 2 × 3 design with repeated measures on the complexity factor was chosen. Subjects were randomly assigned to one of the three assistance systems (AR-glasses, tablet, and paper) and had to assemble four different models of two different complexity levels (low/high). For mental workload operationalization, HR, HRV (rrHRV, RMSSD), PR, FD, SPV, assembly time, and assembly failures were selected. In addition, an AOI analysis was conducted to show potential differences in gaze behavior when assistance systems are integrated in the assembly situation.

### 2.2. Stimulus Material

During the experiment, subjects had to assemble four pneumatic modules taken from the production line of an edge bending machine manufacturer. Models were chosen to represent two levels of complexity based on the number of parts, needed tools, and mounting operations and therefore their individual operator choice complexity [1] as well as the rating of two employees of the edge bending machine manufacturer. For low complexity models, three (M1) or six parts (M2) need to be assembled with two and three degrees of freedom for part orientation (possible failures were classified in wrong parts and wrong orientation). High complexity models had to be built using 16 (M3) or 19 parts (M4) with three or eight parts that had to be in the right orientation.

Assembly instructions were given to the subjects using either a paper, tablet or AR-glasses based approach. The paper instruction needed manual handling to flip pages while the tablet and AR systems were voice controlled. Voice control offered a hands-free solution. While most participants expected that an AI reacted to their commands during the experiment, an investigator was placed in the room next to the working station changing the shown instructional pictures manually.

**Figure 1:**
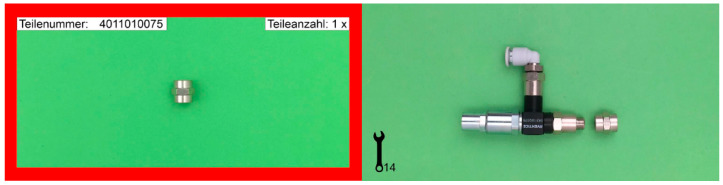
Exemplary assembly instruction (search and assembly parts).

The assembly process was split in two parts: (a) searching and deciding which parts need to be assembled, and (b) a manual procedure of assembling. Instructions were given using the same scheme. In Figure 1, exemplary instructions can be seen for the assembly of a single part to an already existing model. Search parts were marked with a red frame including part code and number of parts while the assembly instruction was based on two pictures, one showing the loose component and the next the assembled result. In addition, the tools to use are shown in the bottom left corner.

### 2.3. Procedure

Subjects were randomly assigned to the assistance system before arrival so the laboratory was prepared for the special circumstances each assistance system needed. Afterward, the arrival of necessary equipment (Holter ECG system, eye-tracking glasses, AR-glasses if necessary) was applied. The main part of the experiment took place in a separate laboratory room without windows and steady light conditions to not influence the pupillary response through lighting changes.

**Figure 2:**
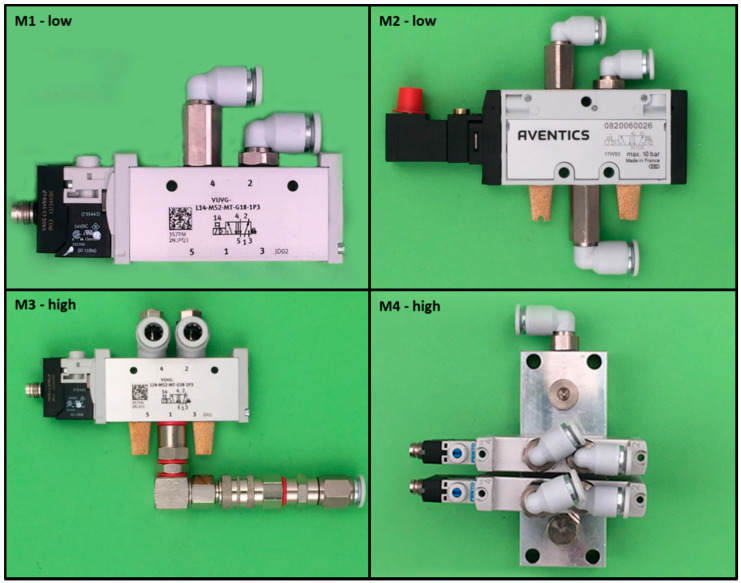
Overview assembly models (M1 and M2 low complexity, M3 and M4 high complexity).

The assembly working station was an exact replica from a real industrial working place with a reduced number of parts and tools. A corner assembly table with two work areas and three rows for parts (all in orange boxes with a unique identification code based on a ten-digit numerical code) were chosen for the setup. The experiment started with an introduction into the assistance system. Participants had to use the system to pick a part and add it to an already preassembled test-model (Figure 1). When they successfully completed this task, they started with the assembly of four models of increasing complexity starting with the low complexity models (M1 and M2) and ending with high complexity ones (M3 and M4) (Figure 2).

All instructions were very detailed in step-by-step instructions. Between the four models, repeatedly used parts, tools, and assembly strategies had to be utilized, but each model also covered new parts and mounting options (like the use of a new tool, introduction of new parts, and therefore a higher decision density).

Based on the experience that complexity has its full detrimental potential on mental workload under time pressure, a gamification approach was chosen to create time-pressure and social competition. On a separate assembly tablet (no assistance system, just for general instructions and gamification), they were always able to see how fast they were at the moment and which place on the leaderboard they had. Higher places on the leaderboard led to an increased chance of winning a gift voucher. Each time they lost a place, an acoustic signal was presented.

After all models were assembled, subjects had to perform a HR baseline measurement sitting still and upright on a chair for five minutes straight.

### 2.4. Participants

Participants (n = 65) were between 18 and 30 years old (mean = 23.48, SD = 3.36) and mainly students. Thirty-nine participants were female (60%). Participants with known cardiovascular diseases, diabetes, and/or epilepsy were excluded from the study. If necessary, participants had to wear contact lenses to correct their sight as the chosen equipment was not able to support glasses.

All participants were informed about the study and gave written informed consent for their participation. The experiment was approved by the ethics committee of the University of Greifswald (Identifier: BB 171/17).

### 2.5. Methods and Apparatus for Mental Workload Assessment

#### 2.5.1. Assistance Systems

The technology behind the used assistance systems were an Apple iPad Air 2 for the tablet and a Vuzix M300 with XpertEye software solution for AR-glasses. The chosen mounting option for the Vuzix M300 supported the parallel usage of AR- and eye-tracking glasses.

#### 2.5.2. Mental Workload Assessment

The assessment of mental workload was based on three indicator groups: performance data, ECG, and eye-tracking. Performance data were recorded in two ways: assembly time was tracked using the assembly tablet where subjects had to acknowledge the start and end of each model and assembly failures were recorded after the experiment using a protocol to check for missing or wrong parts and part orientation.

In accordance to the guidelines for HR and HRV measurement in occupational science [57] for ECG measurement, a 1-channel Faros eMotion 180° Holter ECG with 1000 Hz sampling frequency was used. Eye-tracking data were recorded using SensoMotoric Instruments (SMI) Eye-Tracking Glasses 2 Wireless. Sampling frequency for binocular recording was 60 Hz using the inbuilt infrared sensors.

#### 2.5.3. Data Analysis and Statistics

Data preparation and analysis were performed using Mathworks MATLAB 2019a and the HRV Tool [58] for ECG data and SMIs BeGaze for eye-tracking data. IBM SPSS 25 was used for statistical analysis.

Using the raw EDF-ECG file export of the Holter ECG device and the HRV Tool in-house R-peak detection algorithm based on dynamic thresholds and moving windows [59], the first R-peak detection was performed. Afterward, a manual correction of missing or misplaced R-peaks as well as the deletion of R-peaks detected in highly noisy parts of each ECG dataset was applied. Due to those missing beats, the probability of over- or underestimating the frequency domain HRV parameters increases [60]. Thus, only time domain HRV calculations were chosen using RMSSD and rrHRV [41]. Pupillary response data from both eyes were checked for missing data and outliers and linear interpolation was performed. For further analysis, data from both eyes were averaged to a single indicator.

For all model based analyses, parameter calculations were performed over the total assembly time of each participant and model. To derivate an estimation of MWL, over- and underload reference values over the whole assembly time were determined (mean and standard deviation). For each model, the total percentage of time HR, and PR one SD over or under the mean was calculated. For HR analysis, a continuous calculation approach was chosen (calculating HR over the last 20 heart beats with a 75% temporal overlap).

## 3. Results

To test hypothesis 1 to 3, repeated measures ANOVAs were performed (with complexity as a within and use of assistance system as a between subjects factor). For a further understanding of the achieved results, a manipulation check on subjective complexity perception and gender influence on assembly time was performed.

### 3.1. Manipulation Check

Every two assembled models (two of low and two of high complexity) subjects were asked to rate the perceived complexity on a scale from 1 to 100. There was a significant difference of perceived complexity between the conditions (26.59 vs. 53.52, F(1,60) = 126.762, *p*-value < 0.001, partial η^2^ = 0.679) with no influence of the used assistance system (F(2,60) = 0.216, *p*-value = 0.807).

Due to the randomized assignment of subjects to assistance systems, there was no balanced gender proportion (AR: 12:10, tablet: 15:7, paper: 12:9). Additionally, a repeated measures ANOVA showed no significant differences between female and male participants in assembly times over all models (F(1,63) = 0.001, *p*-value = 0.971) (Table 1).

To further check if there were group specific conspicuities or differences in ECG related parameters, a group comparison of baseline values for HR, rrHRV, and RMSSD was performed. There were no significant differences during baseline measurement between the assistance systems (*p*-values ANOVA HR = 0.215, rrHRV = 0.932, RMSSD = 0.328).

### 3.2. Performance Measurements: Time and Failure

Assembly times and number of failures differed between models with a strong increase from M2 to M3 and smaller differences between models of each complexity level (1 and 2; 3 and 4). While no significant effects could be observed for assembly for the used assistance systems’ time, there was an effect for assembly failures (F(2,62) = 4.683, *p*-value = 0.013, partial η^2^ = 0.131). Planned comparisons revealed that differences between assistance systems did not exist for low complexity (models 1 and 2), but for those with high complexity. The use of a paper based assistance system led to the fewest mistakes, followed by tablet and AR. When differentiated in failures resulting from wrongly used parts and wrong part orientation, no significant differences could be found for used parts but for orientation, which can be seen as an indicator of careful task execution.

### 3.3. Physiological Reactions: Heart Rate and Heart Rate Variability

Mental workload based changes in physiological parameters were analyzed using ECG related parameters (HR, rrHRV, RMSSD) and eye-tracking related ones (PR, FD, SPV). Additionally, the more gaze related approach of AOI analysis was conducted to gain better insight into the usage of various areas at the assembly station.

All chosen mental workload parameters derived from ECG were able to show differences between the different models (Table 2). While they differed in effect size (with the strongest effect size for rrHRV with partial η^2^ = 0.368), they all indicated that subjects in either group were the least stressed during the assembly of M2. While rrHRV was able to differentiate between models in more detail (especially for AR and paper), only RMSSD showed differences between M2 and the remaining models.

In an attempt to quantify limits for individual mental over- and underload, all HR values greater than one SD over the mean (over all models) were defined as overload and one SD below the mean as underload. Using this working definition, it is possible to get a better understanding of the dynamical nature of mental workload. To classify those changes in MWL, the percentage of time in each state was used as an indicator (Table 3). Overall, the decrease in underload over all models was stronger than the increase of overload. M2 again showed the least percentage of working time in the overload area and the most time in the underload area. While AR was the assistance system with the least amount of mental overload during M1 (10.45%), the amount of time in the zone of overload increased to 21.62% for M4, making it the assistance system connected with the highest percentage of overload time.

### 3.4. Physiological Reactions: Pupillary Response, Fixation Duration, and Saccadic Peak Velocity

Eye and gaze related parameters showed a less stringent picture compared to the ECG ones. PR, FD, and SPV were all able to differentiate between models and showed significant interaction effects between complexity and used assistance system. Furthermore, SPV was able to detect a significant effect between the used systems (Table 4).

PR showed similar courses for tablet and paper with an increase from M1 to M2 followed by a decrease for the models with higher complexity. Thus, PR indicates a different reaction pattern than the ECG related parameters. For subjects using AR-glasses, the PR increase continued to M3 and showed a strong decrease for M4. For FD, similar reaction patterns could be seen for AR and tablet following an increase, decrease, increase pattern between models with a strong increase for AR between M3 and 4. For paper, a constant increase up to M3 could be seen followed by a decrease.

For SPV subjects, using the paper based assistance system showed a mirrored course in comparison to the AR and tablet group so the detected differences appeared between paper and AR/tablet with greater differences between M1, and 2, 4, and close to zero differences during M3.

Using the percentage of PR over and under one SD in correspondence to the mean similar results to HR can be seen. Overload decreased from M1 to M2, followed by a steady increase until it reached M4 (Table 5). There was a large difference between the used assistance systems for M1 with AR being the system with over 1/3 of the time, causing subjects to be in a state of overload. For M4, AR still reached the highest values, but it was closer to the tablet and paper solution. For underload, a similar course was observable: there was an increase for underload from M1 to M2 followed by a decrease to M4. This can be assumed that, especially for AR, the first time use of the assistance systems caused additional mental workload and that there was an increase in workload through increasing complexity over Models 2, 3, and 4.

### 3.5. Area of Interest Analysis

**Figure 3:**
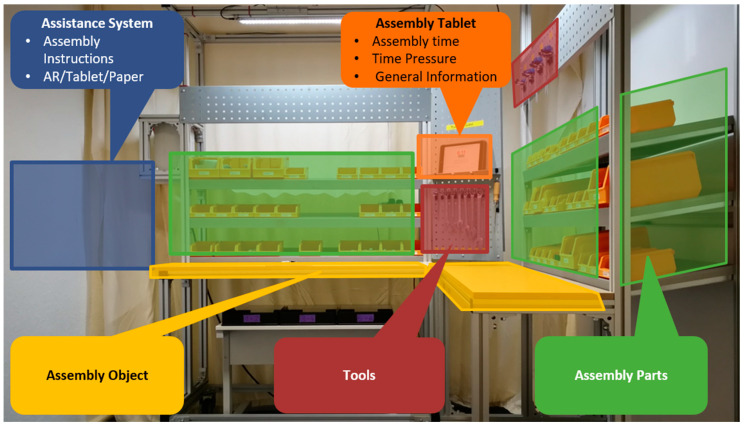
Overview of areas of interest.

Manual assembly is characterized through constant changes between more physical and more mental process parts. Gaze analysis can be used to better understand these dynamics. Figure 3 shows the working space used in the experiment. For the AOI analysis, relevant areas were the used assistance system, assembly parts, and the assembly object as well as the used tools and the assembly tablet (showing assembly time and general information).

**Figure 4:**
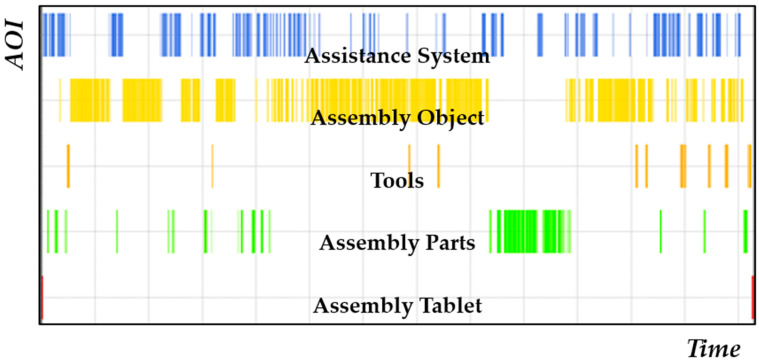
Areas of interest (AOI) over time when assembling Model 4 (only one participant).

Taking a look at the gaze behavior of one participant over the construction time of one model, the dynamic changes can be easily spotted. There was a constant change between fixations of the assistance system, the assembly parts, and the assembly object (Figure 4). The assembly tablet was the least fixated AOI, and is normally only fixated during the start and ending phases of each model. Mentally more demanding process parts are closely connected to fixations of the assistance systems. Participants obtained all necessary assembly information using this system. Depending on the difficulty of the mounting option of a specific assembly part, there is either a longer fixation of the assembly object or shorter revisits of the assistance system to check the information. Longer times fixating the assembly parts usually indicate problems in finding specific parts. Participants used a mixture of object-based search strategies (if assembly objects had a specific form/surface) and searching for the identification number (mainly if the necessary part had many similar looking alternatives).

Using data from 59 participants (six participants had to be excluded due to insufficient tracking rates), an AOI analysis was conducted comparing M1 of low and M4 of high complexity as well as the different assistance systems (Table 6). View distribution was as expected with most views on assistance system (25.85% of dwell time), assembly object (23.93% of dwell time), and assembly parts (19.81% of dwell time) for M1. Due to an overall shorter assembly time and the novelty of the situation, there was a high dwell time percentage for the assembly tablet of 4.83% compared to M4 with only 0.17%. Between M1 and M4, a shift toward the assembly object was observable. Participants spent less time searching for parts (9.85%) and looking up information (assistance system 20.46%), and mostly fixating the assembly object (48.63%).

There was a significant difference between the average fixation duration between the different AOIs (exemplary for M1 F(4,56) = 104.29, *p*-value < 0.001, partial η^2^ = 0.651) with the longest fixations for the assembly object (387.98 ms) and shortest for the tools (177.02 ms) and assembly parts (199.08 ms). The overall scheme did not change between M1 and M4, but there was a slight (close to significant) increase of the average fixation duration for the assistance system (F(1,56) = 3.98, *p*-value = 0.051, partial η^2^ = 0.066) and a slight decrease for the assembly object (F(1,56) = 2.46, *p*-value = 0.122, partial η^2^ = 0.042). For the remaining AOIs, there were no noticeable differences in average fixation time.

To gain further insights into the possible differences in the usage of the assistance systems’ average fixation duration, the fixation percentage per AOI, and number of revisits were compared for both models using a repeated measures ANOVA with the used assistance system as a between subjects factor. There was a significant effect for the used assistance systems on the average fixation duration (F(2,56) = 7.92, p-value = 0.001, partial η^2^ = 0.220), fixation percentage (F(2,56) = 3.60, *p*-value = 0.034, partial η^2^ = 0.114), but not for the number of revisits (F(2,56) = 2.78, *p*-value = 0.071, partial η^2^ = 0.090). Planned comparison shows that the main difference was between AR/paper and tablet/paper (av. fixation duration) and AR/tablet (fixation percentage). Both indicated that participants looked longer, but overall less often, at the information presented using the AR-glasses (even revisits differed from 74.81 for AR to 88.74 for paper during M4.).

## 4. Discussion

### 4.1. General Discussion

The overall findings largely confirm the hypotheses. The assumption of significantly increasing mental workload with high complexity was confirmed, although the factual increases and decreases in the ECG indicators were not particularly pronounced. At the same time, HRV indicators dropped significantly. The increase of choices and corresponding information input not only led to relatively more physical activity and longer assembly times, but also to increased cognitive activity, which in theory contributes to increased sympathetic activity via CNS–ANS integration and its disinhibitory effect. This finding, which has been shown in numerous other research projects [30,31,36], has also received further confirmation and clarification via the eye-tracking indicators. There was an expected increase of fixation duration, which describes the temporal extension of specific information extractions during working on a task. This is also indicated by an additional interaction effect with the assistance system, which is primarily due to the opposite tendencies in the processing of M3 and M4. Only pupil dilation in AR-glasses showed a slight linear increase from M1 to M3 followed by a decrease. Paper and tablet, however, remained at a relatively constant level with no clear trends. This means that the information content and thus the MWL initially increased slightly, but then remained relatively constant at one level in the following models. Subjects have presumably become familiar with the type of task and the associated choice making, search, and assembly activities over the first two models, which reduces the need for additional information extraction. A comparably ambiguous finding can be seen with regard to SPV. There were no significant changes with increasing complexity, whereby interaction effects with the assistance system were evident again.

The examination of AOIs provides additional information on the cognitive activities when subjects concentrate (e.g., on an aspect of the instruction or the object to be assembled). Each gaze activity has the aim to extract information and to initiate manual assembly activities. In this sense, McCarley and Kramer [43] refer to a close coupling of eye movements and object-related actions that bring the targeted action closer to the goal with the smallest steps. The eye and corresponding hand movements that occur during assembly are not random, but follow very different strategies for integrating information acquisition and behavioral performance [61]. For example, it seems strategically sensible to first take note of the instructions, then search for parts to be installed, remove them from the container, and finally assemble it, then turn back to the instruction, check the progress, and prepare the next step. The eye movements thus support forward-facing mounting, and backward-looking fixations (in this study revisits to the assistance system) primarily serve as self-confirmations.

The used assistance systems had no independent beneficial or reducing effect on MWL. They do not affect the execution of tasks with low or high complexity. Performance results are solely determined by the quality of the instructions. Thus, it is the content but not the form of presentation that is decisive for efficient working. This finding should not hide the fact that in practice, instruction maintenance is often neglected [56], probably due to a widespread mindset that assembly work is largely based on routine and cognitive automation. Only when tasks become more demanding and ask for specific voluntary choices during the assembly process–Ballard et al. [61] speak of so-called what/where modules–should support by assistance systems be offered. Individual interaction effects of the model and the assistance system are presumably due to special features of the AR-glasses, which necessitate a higher adjustment effort.

### 4.2. Limitations

Cognitive neuroergonomics has two goals: first, to use existing and emerging knowledge of brain function to design technologies and work conditions for safer and more efficient operation, and second, to advance understanding of brain function in relation to real-world tasks and everyday work [25]. Thus, a major aim of this approach was to validly identify conditions of high cognitive load or mental overload at the real work places and thus to indicate starting points for ergonomic countermeasures. Although our findings support the assumption that more complex tasks lead to higher MWL, we have to realize various inconsistencies in the data. Thus, doubts concerning the view of the MWL construct and its operationalization via different indicators in relation to the fulfillment of natural tasks outside the laboratory have arisen. Many natural tasks used in the laboratory are still close to very narrow experimental paradigms of cognitive psychology [62], to the simulation of greatly reduced driving situations [63], or to the detection of situation embedded, salient stimuli on a monitor [18]. Three central limitations of this study at hand but also of the general approach should be discussed: the relation to the everyday work tasks, the concept of complexity, and MWL measurement using various-related-indicators, leading to the question whether MWL is a unitary or a multidimensional construct [18,64].

In the present study, the intention was to reproduce a real-life assembly activity as a natural task in the laboratory. However, associated boundary conditions of an operational assembly activity could not be optimally transferred to a laboratory task. This applies, in particular, to aspects of the qualification and assembly experience of the employees (respectively subjects), but also to the organization of work and commitment to the task. From a cognitive ergonomic point of view, our subjects did not have a sufficient mental model of the assembly work imposed on them [17]. For example, an attempt was made to operationalize temporal restrictions of real tasks by using a gamification arrangement, but could not actually be enforced stringently.

This missing of effective time limits made subjects rather free to choose their own speed to exert their task. At the same time, this jeopardizes a central assumption of the study. Complexity cannot be conceptualized by solely increasing the number of choices but only in connection with a specified time pressure. Both the number of choices and time pressure are necessary conditions to increase the MWL. If subjects have as much time as they want to select and search parts, complexity disappears. Those who can take any amount of time during assembly do not experience uncertainty or an acute burden. In addition, the lack of experience with assembly activities implies the need to process instructions more intensively, especially at the beginning. This may result in an unexpectedly high MWL during the assembly of M1. After completion of M1, phases of information extraction became shorter due to practice and learning effects over the repeated assembly tasks. This in turn counteracts the assumption that increasing complexity in models 3 and 4 leads to more information extraction and more MWL. Supplementary, for paper- and tablet-based systems, mental models already exist whereas AR-glasses are still new and therefore presumably require more effort and longer familiarization time.

Taking these considerations into account, our operationalization of complexity alone via the number of choices in restricted time showed a further shortcoming. It does not consider the fact that there are dependencies between sequences of choices, which ultimately—and this is shown by the AOI protocols–lead to a different interaction with the information presenting assistance system. From model to model, subjects concentrated their attention more and more on the assembly object. In the course of a concrete assembly, complexity and uncertainty decrease with the reduction of degrees of freedom due to the restricted possibilities to add further parts and give room for more intuitive actions [65]. In addition, learning processes reduce search times for parts and facilitate the selection of tools. All of these aspects of a natural task contribute to the fact that the complexity of the task was continuously reduced by repetition [66]. Taken together, natural tasks such as assembly or other manual work should not be seen as static individual action but as a dynamic variable over a period of time. The question arises whether and to what extent natural tasks fulfill the experimental presumptions of a stable and interindividual invariant entity.

A final aspect concerns the measurement of varying MWL that was carried out using ECG and eye-tracking indicators. This combination corresponds to recommendations to not focus on a single indicator solution of an assumed multimodal construct like MWL [30,32]. Both groups of indicators we used relate to very different organismic systems which, however, experience central control prefrontal areas of the cortex [37]. This might support a more unitary view of MWL. In contrast, the group of ECG indicators describes relatively slowly developing changes while the eye related ones showed quicker adaption processes, although both were initialized via the same autonomic nervous system. Thus, there probably is no simultaneously organized activity to be measured. This might favor a view of a more multidimensional approach. Due to improved measurement technology, we are able to measure rather precisely the fixation times or pupil dilations as well as R-peaks and HRV data. However, it is difficult to relate these data to one another for a defined point or period in which mental stress and overload could have occurred. An important prerequisite for a multidimensional measurement of MWL could be to coordinate the spatio-temporal sensitivity of both measures and to show that there is some common variance. Our discrepancies between ECG and eye-tracking data raise doubts about the idea of a unitary concept.

The dimensionality debate is primarily theoretical. For the practical ergonomist, the main question is how to calculate a person’s MWL with regard to a task. For practical purposes, it is not enough to theoretically accept a redline you have to be able to determine how pronounced the MWL is and where there are lower and upper limits that should not be exceeded. There is a lack of possibilities to determine such absolute extents of MWL and normative limits [10]. For this reason, comparisons between conditions or groups of people predominate in research. This is an important field of work for the future. In a multidimensional perspective, this indicates determining the several limits of different measurement techniques and indicators. At the same time, standards would have to be developed for each measurement procedure, particularly if it is used in the context of natural tasks.

A more practical concern might be the influence of the surrounding on pupil size during the experiment. Even though pupillary response has shown to be sensitive for changes in MWL, this reactivity to changing states of workload and arousal is only one of three underlying mechanisms that induce changes of pupil size [48]. The remaining two are the pupil light reflex and the pupil near or accommodation reflex, of which both could have an influence on this study. More research has been done on the pupil light reflex focusing on changes of pupil size due to changing light conditions, resulting in a pupillary constriction for increases in brightness and vice versa, a dilation for increasing darkness [67]. During the experiment, we focused on keeping lighting conditions constant. Therefore, three sources of light were applied on the assembly station, two directly above each working area (see assembly object in Figure 3) and one central over the assembly station at the ceiling. Thus, the room should have been evenly illuminated. Even with promising approaches to eliminate the influence of light on pupillary response during mental workload, further research and additional light sensors are needed to use them [68]. While lighting and brightness should not be an issue for the used assistance systems (tablet was set to a medium brightness for good visibility, but no disturbing brightness), the pupil near reflex could have an influence on the comparison due to decreased distance for the information available on the AR-glasses. The pupil near reflex “is certainly the least studied, and perhaps the least understood of all pupil responses” [48] (p. 10). It describes the dilation of the pupil focusing on distant objects and the constriction focusing on near ones. During the experiment, most visual cues were presented in a range of 1.5 m. For AR-glasses, an optical see-through version was chosen with a small projection area in the top right corner that did not interfere too much with the user’s natural field of view [69]. Thus users of the AR-glasses could have an overall smaller pupil size due to the shorter fixation distance for the presented instructions. Noticeable differences only occurred during M1 with 3.29 mm against 3.43 mm (Tablet) and 3.54 mm (Paper) (Table 4). Still, the participants in this group showed the highest amount of overload during M1 (34.53%, Table 5), therefore the pupil near reflex seems to be a negotiable issue for this study.

Including EEG and neuroimaging techniques might be a way to obtain a closer look at the brain at work, but will also be more intrusive and lead to additional questions like how to handle latency between different techniques, which signals refer to task specific changes and which ones to wandering thoughts, and how to handle the comparatively low number of task repetitions in a more field-like environment.

## 5. Conclusions

The program of cognitive neuroergonomics is strongly directed toward providing recommendations for ergonomics based on neurological principles. For Parasuraman [25], this was a dream of the future. As he saw it, the brain interacts with the world via a physical body, which is why neuroergonomics should be concerned with the neural basis of each kind of physical activity at work and in even more complex contexts of everyday life. Thus, the neurocognitive approach allows us to ask different questions and develop new explanatory frameworks about work, which traditional ergonomic approaches based primarily on the measurement of overt performance and subjective impressions cannot ask. Aside from serious voices that generally cast doubt on the approach [15], such a position faces many challenges. As we wanted to show, there are still considerable theoretical and practical measurement problems, especially when it takes place outside the laboratory in the natural environment. Followers of cognitive neuroergonomics are optimistic that there are opportunities in the future to identify cortical stress conditions in real time with algorithmic support and to combine them with ergonomic countermeasures. This is still a long way to go, especially if it should be shown that the functional processes running in the person’s brain are generally dynamic and of higher interpersonal variability.

## Figures and Tables

**Table 1:** Gender based differences in assembly time over all models.

		Model 1	Model 2	Model 3	Model 4
Female	Mean	126.46	164.64	561.33	686.97
(*n* = 39)	SD	24.74	40.93	115.48	143.09
Male	Mean	129.89	165.48	567.22	675.15
(*n* = 27)	SD	30.21	49.97	190.12	165.73
overall	Mean	127.86	164.98	563.74	682.14
(*n* = 66)	SD	26.94	44.48	149.21	151.63

**Table 2:** Electrocardiogram related mental workload parameters.

		Low Complexity		High Complexity				
		Model 1	Model 2	Model 3	Model 4	Models	Assistance System	Interaction
HR	AR	93.71	92.52	95.16	96.17	0.158 ***	n.s.	n.s.
	Tablet	97.50	95.34	96.98	97.29			
	Paper	93.02	91.22	93.69	93.73			
rrHRV	AR	3.71	3.95	3.47	3.19	0.368 ***	n.s.	n.s.
	Tablet	2.83	3.07	2.89	2.78			
	Paper	3.67	4.06	3.64	3.46			
RMSSD	AR	28.79	30.39	27.95	25.17	0.100 **	n.s.	n.s.
	Tablet	20.87	22.62	21.02	21.95			
	Paper	28.73	33.75	28.78	27.61			

α-level ** = 0.01, *** = 0.001; n.s. = non-significant.

**Table 3:** Quantification of over- and underload using heart rate (% of time one SD over/under mean).

		Model 1	Model 2	Model 3	Model 4
AR	Underload	20.04	28.02	11.26	9.45
	Overload	10.45	12.30	14.21	21.62
Tablet	Underload	9.97	21.06	9.79	11.73
	Overload	20.21	14.52	15.38	17.21
Paper	Underload	16.37	23.27	10.27	12.21
	Overload	16.08	11.47	16.11	19.97
Overall	Underload	15.44	24.13	10.44	11.11
	Overload	15.57	12.79	15.22	19.59

**Table 4:** Eye and gaze related mental workload parameters.

		Low Complexity		High Complexity				
		Model 1	Model 2	Model 3	Model 4	Model	Assistance System	Interaction
PR	AR	3.29	3.46	3.58	3.41	0.113 ***	n.s.	0.095 **
	Tablet	3.43	3.52	3.44	3.42			
	Paper	3.54	3.66	3.58	3.53			
FD	AR	224.01	240.03	217.38	258.64	0.166 ***	n.s.	0.184 ***
	Tablet	212.03	233.22	223.08	234.52			
	Paper	213.89	217.79	234.00	221.79			
SPV	AR	167.66	166.91	180.89	151.90	0.053 **	0.203 **	0.080 ***
	Tablet	182.63	167.19	182.09	165.04			
	Paper	212.08	209.43	188.87	198.35			

α-level ** = 0.01, *** = 0.001; n.s. = non-significant.

**Table 5:** Quantification of over- and underload using pupillary response (% of time one SD over/under mean).

		Model 1	Model 2	Model 3	Model 4
AR	Underload	10.57	18.20	11.15	13.68
	Overload	34.53	6.16	9.57	17.46
Tablet	Underload	19.74	25.03	14.33	12.53
	Overload	10.05	6.34	8.62	12.49
Paper	Underload	16.78	24.52	16.65	9.03
	Overload	13.19	4.83	8.07	15.82
Overall	Underload	15.48	22.43	13.98	11.75
	Overload	19.80	5.76	8.77	15.38

**Table 6:** Areas of interest comparison of Model 1 and Model 4 (mean values).

		Assistance System			Assembly Object			Assembly Parts			Tools			Assembly Tablet		
		AR	Tablet	Paper	AR	Tablet	Paper	AR	Tablet	Paper	AR	Tablet	Paper	AR	Tablet	Paper
M1	Dwell Time (s)	34.93	33.07	34.43	29.82	28.94	36.33	28.65	26.80	25.58	3.68	2.37	2.54	8.58	7.00	6.13
	Dwell Time (%)	25.05	26.26	26.32	20.76	23.32	28.05	19.57	21.05	18.84	2.14	1.32	1.58	4.95	5.21	4.32
	Av. Fixation Duration (ms)	248.05	238.37	208.79	387.67	393.32	383.00	189.52	203.11	205.63	178.05	189.47	163.42	236.43	247.42	246.37
	Fixations	112.57	112.47	125.47	67.43	66.16	84.47	108.62	91.95	86.16	14.24	8.74	10.37	28.86	23.42	20.42
	Fixations (%)	32.41	37.56	38.60	22.96	22.05	26.00	31.31	29.64	25.55	4.42	2.86	3.21	8.43	7.74	6.26
	Revisits	15.86	17.05	17.89	8.19	10.47	11.53	7.29	6.11	5.74	2.71	1.58	1.63	3.38	3.11	2.63
M4	Dwell Time (s)	61.93	65.54	65.54	65.54	65.54	65.54	59.53	55.87	56.05	11.27	9.42	10.33	4.16	5.57	3.94
	Dwell Time (%)	19.86	20.63	20.95	46.67	48.16	51.26	10.19	10.37	8.95	1.24	1.11	0.95	0.10	0.37	0.05
	Av. Fixation Duration (ms)	262.57	249.84	214.16	374.29	373.47	372.63	193.52	203.26	204.00	188.19	193.84	187.21	235.33	293.42	301.53
	Fixations	437.24	420.68	536.89	815.19	702.05	879.53	269.10	225.84	232.32	42.62	36.00	39.21	14.90	17.58	11.89
	Fixations (%)	27.75	30.17	32.19	50.86	49.24	50.76	17.33	16.42	13.70	2.74	2.65	2.31	0.94	1.22	0.73
	Revisits	74.81	77.42	88.74	64.14	65.11	78.00	28.05	20.53	22.53	10.43	8.21	8.89	3.38	3.21	2.79

Sample size: AR = 21, tablet = 19, paper = 19.

## Data Availability

The data presented in this study are available on request from the corresponding author.
